# Compliance with Nutritional and Lifestyle Recommendations in 13,000 Patients with a Cardiometabolic Disease from the Nutrinet-Santé Study

**DOI:** 10.3390/nu9060546

**Published:** 2017-05-26

**Authors:** Solia Adriouch, Hélène Lelong, Emmanuelle Kesse-Guyot, Julia Baudry, Aurélie Lampuré, Pilar Galan, Serge Hercberg, Mathilde Touvier, Léopold K. Fezeu

**Affiliations:** 1 Université Paris 13, Equipe de Recherche en Epidémiologie Nutritionnelle (EREN), Centre de Recherche en Epidémiologie et Statistiques, Inserm (U1153), Inra (U1125), Cnam, COMUE Sorbonne Paris Cité, F-93017 Bobigny, France; h.lelong@eren.smbh.univ-paris13.fr (H.L.); e.kesse@eren.smbh.univ-paris13.fr (E.K.-G.); j.baudry@eren.smbh.univ-paris13.fr (J.B.); a.lampure@eren.smbh.univ-paris13.fr (A.L.); p.galan@eren.smbh.univ-paris13.fr (P.G.); s.hercberg@eren.smbh.univ-paris13.fr (S.H.); m.touvier@eren.smbh.univ-paris13.fr (M.T.); l.fezeu@eren.smbh.univ-paris13.fr (L.K.F.); 2 Département de Santé Publique, Hôpital Avicenne, F-93017 Bobigny, France

**Keywords:** dietary quality, dietary recommendation, cardiometabolic diseases

## Abstract

Background: A healthy diet has been shown to prevent cardiovascular diseases complications. The objective of this study was to assess dietary intakes and compliance with nutritional and lifestyle recommendations in French adults diagnosed with hypertension, diabetes, dyslipidaemia or cardiovascular disease compared with healthy individuals. Methods: Data was collected from 26,570 subjects aged 35 to 70 years (13,285 patients and 13,285 controls matched by sex and age) of the French cohort NutriNet-Santé. Dietary intakes were assessed using three 24-h records. Mean food and nutrient intakes of patients were compared to those of healthy subjects using multivariable mixed logistic and linear regressions. Results: Compared to healthy controls, adults reporting cardiometabolic diseases had lower intakes of sweetened products, higher intakes of fish and seafood and a better compliance with dairy products. However, overall, they reported unhealthier lifestyles and dietary habits. Indeed, they were less often physically active and had similar habits regarding alcohol and tobacco consumption. They also had lower intakes of fruit, higher intakes of meat, processed meat and added fats. It is noteworthy that diabetic subjects tended to show the highest compliance with certain dietary recommendations (vegetables, pulses and whole grain products). Conclusion: Our study brings into focus the fact that some nutritional aspects still need to be improved among individuals with a cardiometabolic disease. We should encourage higher intakes of fruits and vegetables, whole grain products, and lower intakes of meat and sodium, as well as healthy lifestyle (physical activity, no-smoking and limited intake of alcohol) in order to encourage a healthier management after being diagnosed.

## 1. Introduction

Cardiovascular diseases (CVD) are the major causes of worldwide mortality [1]. In Europe, CVDs are the most common cause of death, and in France they account for one third of deaths in men and one quarter of deaths in women. Improvement in the management of CVDs over recent decades has resulted in an increase in the number of patients living with these diseases, leading to long-term management of chronic diseases in a population with higher life expectancy.

Over the past decades, secondary preventive measures have greatly improved, regarding the medical follow-up of patients. Data from an extensive number of randomized trials and meta-analyses have demonstrated that such measures foster healthy behaviours, promote active lifestyles and reduce cardiovascular risk and event rates [2,3,4]. Various existing strategies for secondary prevention encompass the management of risk factors such as elevated blood pressure, dyslipidemia, and raised fasting blood glucose, as well as the appropriate prescription and adherence to cardio-protective drugs. They also include comprehensive lifestyle modifications based on behavioural change models (i.e., smoking cessation, healthy food choices, stress management and exercise training), with greater patient involvement in making decisions regarding their illness.

After diagnosis of a cardiometabolic disease (CMD), which in our study includes hypertension, diabetes, dyslipidaemia and CVD, clinical practice guidelines from the European Society of Cardiology and the American Heart Association recommend a higher intake of fruit and vegetables (high in fibres), fish/poultry/nuts, whole grains, and low-fat milk products [2,3,4]. In addition, these guidelines recommend specific restrictions, such as sodium, sugar and sweets, saturated fats, total fat, and refined carbohydrates of processed foods. In France, The ‘Programme national nutrition santé’ (PNNS), the national public health program on nutrition and health, implemented in 2001 by the Ministry of Health has also issued specific recommendations [5] to manage hypertension, diabetes, dyslipidaemia and cardiovascular events, in addition to the simple and well-known general recommendations aimed at the general population but the dietary recommendations are the same for the general population and depend on individuals' personal medical monitoring.

However, according to the results of the recent PURE study [6,7], optimal medical treatment, including referral to a cardiac rehabilitation after a cardiovascular event, is still under-prescribed worldwide. Besides, recent studies showed that patients adhere less to lifestyle modifications than to their drug regimens one month after acute coronary syndrome and that only a quarter of patients adhere to their drug regimens after myocardial infarction. In France, the situation is similar: the PREVENIR study conducted in 1394 patients in the post-myocardial infarction period or presenting unstable angina showed that, at 6 months, 50% were still current smokers, 66% had blood levels of low density lipoproteins cholesterol higher than the French Agency for the Safety of Health-Care Products (AFSSAPS) recommendations and that 27.4% had non-controlled arterial hypertension [8].

The present study relies on a large population-based survey including about 13,000 patients with a history of CMD from the NutriNet-Santé cohort. Its general aim was to assess dietary intakes (food groups and nutrient intakes, including polyphenols) and compliance with French nutritional and lifestyle recommendations for cardiometabolic patients compared to healthy individuals. This study was not designed to investigate any causal relationships between diet and CMD but to fill gaps in knowledge on dietary habits among patients with specific types of CMD. We assumed that patients would not substantially alter their lifestyle habits after a CMD diagnosis. Thus, the objective of this study was to assess which dietary habits need to be improved in patients with specific types of CMD—in order to provided important information for the design of efficient and well-targeted secondary prevention strategies.

## 2. Methods

### 2.1. Study Population

Participants were taken from a sample of volunteers from the NutriNet-Santé study, a prospective observational cohort study in order to evaluate the relationships between nutrition and health. The NutriNet-Santé study’s aims and methods have been described in details elsewhere [9]. Briefly, participants were included in the cohort once they completed a baseline set of questionnaires assessing dietary intake, physical activity, and socioeconomic and health status. At follow-up, participants completed the same set of questionnaires every year or sixth months. Additionally, each month they were invited to fill out complementary questionnaires related to determinants of food behaviours, nutritional and health status. Informed consent is obtained electronically from all participants. All procedures were approved by the Institutional Review Board of the French Institute for Health and Medical Research (IRB Inserm No. 0000388FWA00005831) and the French National Information and Citizen Freedom Committee “CNIL” (No. 908450 and 909216).

### 2.2. Data Collection

#### Sociodemographic, Lifestyle and Anthropometric Data

Self-administered questionnaires were annually proposed to participants during follow-ups to collect and update data on sociodemographic, lifestyle and behavioural characteristics, including sex, age, geographical region of residence, marital status, number of children, educational level, smoking status, anthropometry and leisure-time physical activity. The latter was estimated using the validated international physical activity questionnaire [10]. All baseline questionnaires were pilot-tested and then compared against traditional assessment methods [11,12].

### 2.3. Dietary Data

At baseline, and every 6 months, participants were invited to complete three non-consecutive validated web-based 24-h dietary records, randomly distributed between week and weekend days to take into account intra-individual variability. Twenty-four-hour dietary records used in this cohort were tested against a traditional interview by a dietitian [13] and validated; they showed good validity when compared with biomarkers of intake [14,15]. Completion was made via a secured user-friendly interface and was designed for self-administration on the Internet. Participants were asked to report all foods and beverages (type and quantity) consumed at each eating occasion. Nutritional values for energy, macronutrients and main micronutrients came from a published nutrient database [16]. Portion sizes were assessed via a validated picture booklet [17] or according to standard measurements.

For each participant, daily nutrient intakes were calculated using the ad hoc NutriNet-Santé food composition table [16]. Polyphenol intakes were assessed by matching this database with the polyphenol content of each reported food from the Phenol-Explorer database [18]. The 15-point PNNS-GS (Tables S1 and S2) is a validated a priori score reflecting the adherence to the official French nutritional recommendations which has been extensively described elsewhere [19]. Details on computation of this score are available in Table 1. Briefly, scoring and cut-off values (Table S1) were decided using information provided by national recommendations, themselves based on epidemiologic and clinical evidence. At least one point was attributed for each component when subject behaviour was in accordance with the recommendation (Tables S1 and S2). For most components, intermediate points were attributed to subjects who did not entirely attain the recommendation but came close. Additional points were attributed when fruit and vegetable intake was >7.5 servings a day, salt intake was <6 g per day and physical activity was >1 h of moderate activity per day. A half point was deducted when added simple sugars from sweetened foods and salt intake were too high, because of their proven association with chronic morbidity. In addition, we also considered specific recommendation for patients issued by the international society or associations for patients (Table S3).

Each component cut-off was that of the threshold defined by the PNNS dietary recommendations corresponding to the public health objectives of the PNNS in the general population [3]. The overall score has a minimum of 0 points and a maximum of 15 points. Theoretically, a high score reflects behaviour that is in accordance with national recommendations. In our study, we considered the mean PNNS-GS score, and the compliance to the French dietary recommendations (proportion in the highest sex-specific quartile of the PNNS-GS score) was taken into consideration. Each proportion of participants complying with the recommendation in Table 2 was estimated by a threshold corresponding to have at least one score equal to the one representing the criteria in Table S1. Compliance with each specific recommendation was defined as obtaining a score of at least 1 for the specific recommendation (according to the scoring system shown in Table S1).

#### 2.3.1. Cases Ascertainment

At baseline, a self-administered questionnaire is used to collect information on personal history of CVD (prevalent cases). Thereafter, health events are regularly self-reported by the participants during the follow up and validated by the medical team based on pathological reports (incident cases) or additional information on other relevant data, such medical treatments declared regarding their illness (prevalent cases). The present study focuses on cases of hypertension, diabetes mellitus (type 1 and 2), dyslipidaemia (hypercholesterolemia and hypertriglyceridemia), and cardiovascular events (strokes, transient ischemic attacks, myocardial infarctions, acute coronary syndromes and angioplasties) upon the inclusion in the NutriNet-Santé study. A large single category of cardiometabolic disorders was defined as “having at least one of the diseases mentioned above”.

#### 2.3.2. Statistical Analyses

Of the total sample of *n* = 117,923 enrolees in the NutriNet-Santé, between May 2009 and May 2016, e-cohort who had completed at least three 24 h-dietary records (and a maximum of 24 h-dietary records) during the first two years of the follow-up, we selected the 75,584 participants aged between 35 and 70 years. Pregnant women (*n* = 430), participants who reported a prevalent cancer (*n* = 5589), energy-under-reporters (*n* = 7429) using the method proposed by Black [20] and incident cases of cardiometabolic diseases during the first two years of the follow-up (*n* = 3711) were excluded, leaving 58,174 participants eligible for the present work. For each participant who declared a cardio metabolic disorder (CMD or CVD) at baseline, and for each category of disorder, one control was randomly selected among the 44,332 subjects without any prevalent or incident CMD and was matched on sex and age (2 y classes). The controls were free from any prevalent CVD, CMD or cancer.

The matching procedure was conducted separately for each of the five categories of CMD described above (random selection with replacement from a category of CMD to another). Our final study sample consisted of a maximum of 13,285 patients with CMD matched with 13,285 controls for the largest category (any CMD). Study participants’ characteristics were compared with paired *t* tests or MacNEmar Chi2 tests, as appropriate, across cases and controls.

Means (dietary intakes) and proportions (adherence to French dietary guidelines) were adjusted for age, sex, energy intake, number of dietary records and season of dietary assessment (spring/summer or autumn/winter).

Adherence to the nutritional recommendations (in term of macronutrients) was evaluated also in terms of percentage to total energy, in Table S4.

For this purpose, the generalized linear mixed-effect model was used to take into account the effect of the randomization, with logit link function for binary outcomes and normal link function for continuous outcomes. The GLIMMIX procedure in SAS software version 9.3 (SAS Institute, Inc., Cary, NC, USA) was used to obtain adjusted means and proportions for lifestyle and nutritional behaviours, making it possible to use non-normal data.

Because the large sample size increases the likelihood of significant statistical tests for very small differences with low biological/clinical relevance, results were interpreted as significant only when *p*-value was <0.01 to avoid the error of multiple testing. We discussed the results only when the relative difference in dietary intakes or adherence to recommendations between cases and controls was >5%. Relative difference in intake between the patients the controls was computed as: ((mean intake of the patients − mean intake of the controls)/(mean intake of the patients)) × 100. A positive relative difference shows higher intake of nutrient in the patients and a negative relative difference shows lower intakes in the patients. Statistical analyses were performed using SAS software (version 9.3, SAS Institute).

## 3. Results

In total, 7801 cases of hypertension, 1759 cases of diabetes mellitus (298 type 1 and 1468 type 2), 7063 cases of dyslipidaemia, 1098 cases of cardiovascular events (506 coronary diseases and 692 strokes), given a total of 13,285 individuals with at least one of these CMD were included in the present study, as well as one matched control for each case (*n* = 26,570).

Characteristics of included participants are presented in Table 1. Mean age of participants ranged from 55.5 (CMD patients) to 56.9 (hypertensive and CVD patients) years old according to the CMD category. Women represented approximately two thirds of the different study samples, except for the diabetes and cardiovascular events samples, where the proportion of women was lower.

Individuals with a CMD had a lower educational level (>2 years after high school degree: 51.7.6% vs. 56.9.4%, *p* < 0.0001) than controls, especially for subjects with hypertension and diabetes. They had also a higher BMI (≥25 kg/m^2^: 31.8% vs. 55.5%, *p* < 0.0001), especially for diabetes subjects (≥ 25kg/m^2^: 32.3% vs. 76.5%, *p* < 0.0001). The participants with diabetes or dyslipidaemia or a CMD were statistically less often married than controls.

Table 2 compares the percentages of patients and their healthy controls adhering to the French nutritional recommendations. For each category of CMD, adherence to physical activity recommendation was lower in patients (by −8.0% to −13.5%) than in controls. No differences were found between patients and controls concerning the recommendations pertaining to smoking and alcohol consumption. Consuming at least five servings daily of fruit and legumes did not differ between patients and controls, except among hypertensive patients who had a lower adherence to this recommendation (−5.3%). The recommendation pertaining to “salt” and “whole grain products” showed the lowest compliance among both groups with a better adherence for controls than patients. Even if no significant difference higher than 5% was found between patients and controls regarding the mean PNNS-GS-Score, hypertensive individuals were less likely to belong to the highest quartile of the score (−12.2% to be in the highest quartile of the PNNS-GS score).

Compared to other patients, individuals with diabetes had the highest adherence to the different recommendations, except for added fats, physical activity and salt. They had better adherence to recommendations regarding sweetened beverages (8.6%) and added simple sugars (14.1%) than non-diabetic patients. Individuals with dyslipidemia had a better adherence to the recommendation concerning consumption of fish and seafood (+8.8%).

Regarding the specific recommendation, in Table S3, the results also indicated that patients did not follow the specific recommendation issued by the international society, except for the total carbohydrates in diabetic patients.

The multivariable comparisons of the different food groups intakes among patients and their healthy controls are presented by category of CMD in Table 3. Across all groups of patients compared to their matched controls, patients consumed more processed meats (from +7.9% in patients with CVD to +23.7% in diabetes patients) and red meat (from +11.3% in patients with CVD to +25.2% in diabetes patients), poultry (from +5.0% in patients with CVD to +21.4% in diabetes patients) and less fruit (from −6.3% in patients with dyslipidaemia to −15.2% in diabetes patients), sweet products (from −7.4% in patients with dyslipidaemia to −53.8% in diabetes patients) and tea (from −12.6% in patients with dyslipidaemia to −45.5% in diabetes patients). There were no statistical differences between cases and controls concerning intakes of alcohol, cheese, starchy foods, and fatty and sweet products. Although hypertensive patients consumed less sugar, they drank more sweetened drinks than non-hypertensive subjects (+12.8%). Diabetic patients consumed fewer sweet products (−53.8%), fatty and sweet pastries (−15.6%), fruits (−15.2%) and more vegetables (+7.5%) and dairy products (apart from cheeses +14.8%) than their controls. Patients with dyslipidaemia consumed fewer eggs (−19.0%) and more fish and seafood than their controls (+7.5%). Hypertensive patients and patients reporting CVD or a CMD had lower intake of whole grain products or pulses than controls. Higher intake of coffee was observed in all CMDs patients, except for patients with a CVD (from +5.8% in patients reporting CMDs to 18.0% in diabetic patients). Vegetable oils and margarines were more consumed by all patients reporting CMDs, except for hypertensive patients (from +6.6% in CMD patients to +12.0% in diabetes patients).

The multivariable comparisons of nutrient intakes in their patients and their matched controls are presented by the category of CMD in Table 4. The largest differences between patients and controls were observed for intakes of total and simple carbohydrates. Notably, diabetic patients consumed fewer carbohydrates (−8.1%) including added simple sugars (−54.0%) and complex carbohydrates (−21.0%) and more polyunsaturated fats (+5.6%), cholesterol (+6.5%), proteins (+9.0%), and sodium (+10.0%) than controls. Patients with dyslipidemia had lower intakes of added lipids sourced from animals (−16.0%), as expressed also in Table S3.

Table 5 shows, for each CMD, the multivariabe adjusted mean intakes of each polyphenol class, in patients and their control counterparts. All patients exhibiting a CMD had lower intakes of catechins (main contributor: tea), teaflavins (main contributor: tea) and anthocyanins (main contributor: fruits and berries), except for patients with dyslipidaemia. Diabetic patients had lower intakes of flavanons (citrus fruits, red wine) and hydroxybenzoic acid (coffee) than controls. Patients with diabetes or dyslipidaemia had higher intakes of hydroxycinnamic acids. Hypertensive patients had lower intakes of flavonols (tea, green vegetables, fruits, onions) than controls.

## 4. Discussion

In this large population-based study, we aimed to characterize and to compare CMD patients and their health-matched controls in terms of dietary intakes and compliances with recommendations regarding diet (including polyphenols), alcohol intake and physical activity.

We observed that patients adopted certain healthy behaviours more thoroughly (lower intake of sweetened products, higher intakes of fish and seafood and better compliance of dairy products recommendation) than controls, while the opposite was observed regarding physical activity, consumption of alcohol, tobacco, fruits, meat and processed meat, added fats, and more specifically, sodium intake in diabetic patients.

Regarding adherence to official French recommendations guidelines, we did not find a better compliance among patients, despite having probably benefited from lifestyle counselling, after the diagnosis. The fact that diet quality in subjects with CMDs was comparable to those with no disease is consistent with the literature as they should have been more aware of a nutrition and healthy lifestyle issues by having to manage their disease. However, it has been previously reported that subjects with CMDs do not meet dietary recommendations. This is particularly true for patients that have experienced cardiovascular events (stroke or myocardial infarction) [21,22,23] and hypertensive patients [24,25]. However, these results need further investigation as design and methods varied. Some studies have shown a better compliance by diabetes patients to official recommendations compared to population controls [26] or among patients who had experienced cardiovascular events. Wallstrom et al. conducted a study among subjects with a history of acute myocardial infarction, using a similar design in which subjects were age-matched with controls [27]. In their study, patients were more in line with current recommendations than controls, particularly regarding the consumption of fat, which was lower. In France, Castetbon et al. have shown that healthier diets of individuals with diabetes after diagnosis did not translate into greater compliance with recommendations based on a nationally representative French population aged from 45 to 74. It would seem that subjects had a wider gap to bridge, and therefore attaining recommendations was more striking than for controls [28] before their diagnosis of diabetes. In our study, we found a similar result regarding profiles of compliance with recommendations in case and control groups. Some differences were, however, highlighted. Although, the prevalence of “current smokers” was almost twice as low in our study sample, compliance with “smoking and alcohol habits” showed no difference between patients and controls and physical activity recommendation was systematically lower in patients. Physical activity, smoking cessation and moderation of alcohol should be improved, since they are major lifestyle factors causing the development of cardiovascular diseases [29]. In contrast, certain specific and additional recommendations in the management of each disease seemed well assimilated. Diabetes patients thus showed better compliance with the recommendation pertaining to sugar products but not in added fats while dyslipidemia patients had a better compliance with the recommendation regarding consumption of fish and seafood. Consumption of whole grain products was the recommendation that was the least followed by our population, which should be more targeted in future studies regarding dietary support on recommendations [30], particularly in diabetic patients who need to control their glucose blood level. In addition, despite differences of adherence in specific recommendation according to each CMDs, the global adherence score to the PNNS was not different between cases and controls.

This may be partly explained by the fact that, overall in CMDs patients, some intakes are in line with a healthy diet and other aspects still need to be improved. However, hypertensive patients were less likely to belong to the highest quartile of the PNNS-GS score, as suggested by previous studies [24,25,26,27,28,29,30,31].

Regarding micronutrient and macronutrient intakes, results of diabetes patients and controls were more different from one another. Diabetes patients consumed fewer carbohydrates and simple sugars, more polyunsaturated fats and proteins while they had higher intakes of cholesterol and salt. This may be explained by their higher consumption of processed meat, red meat and poultry and lower consumption of fruits. Castetbon et al. showed that diabetes patients had higher intakes of proteins and lower intakes of sugars and total carbohydrates while they consumed fewer calories and globally exhibited a healthier diet [32]. Many studies have shown that individuals with diabetes consumed more proteins [33,34,35], meats [32,33,34,35,36], sodium [37,38] and also more fat, saturated, monounsaturated and polyunsaturated fat and cholesterol and lower sugars, than individuals without diabetes [21,22,35,36,37,38,39], which is consistent with our findings. In contrast, in our study, the higher consumption of fat was mainly from polyunsaturated fat. This could be considered as a good practice and can be a replacement for saturated fat [40]. Regarding fruits and legumes, unlike the previous studies [21,22,23,41,42], consumption of fruit was lower among subjects exhibiting all categories of diseases. We however found a similar overall compliance of dietary recommendations between cases and controls, except for hypertensive patients.

High consumption of some polyphenols has been associated with a reduced risk of hypertension, type 2 diabetes and cardiovascular diseases [43,44]. The lower intake of anthocyanins, catechins, theaflavins, flavonols, and hydroxybenzoic acids in individuals with CMDs could be explained by their lower intake of fruit and tea, since non-alcoholic beverages, red wine and fruit were the most important contributors to polyphenol intakes [45] and subjects exhibiting all categories of CMDs in our study had a lower consumption of tea and fruit. The higher intake of hydroxycinnamic acids in dyslipidaemia and diabetes patients was mainly due to their higher intake of coffee. In spite of their beneficial effects on cardiovascular health, it is too early to establish dietary recommendations on polyphenol intake.

Regarding the dietary and lifestyle patient education of individuals with CMD, it has already been historically initiated on diabetics. Our results also highlight that particular effort should be made in CVD and hypertensive patients, as suggested in previous studies [46,47,48]. Indeed, dietary and lifestyle education are potential strategies regarding prevention of CVD, and strategies to lose weight, especially because patients were more obese than controls in our study [49].

The strengths of this study include the high number of patients with CMD using an age-sex matched design and detailed and precise dietary and lifestyle data. Indeed, quantitative dietary data was accurately assessed via repeated three non-consecutive 24-h dietary records, repeated every six months, accounting for intra-individual day to day and seasonal variability. Some limitations should be acknowledged. First, subjects included in the NutriNet-Santé cohort were volunteers involved in a long-term study on nutrition and health. Therefore, as it is often the case in health-related cohorts, they were probably more health conscious, and therefore may have had healthier diets and practices compared to other patients in the general population. Secondly, CMDs in this study were self-reported, although they were validated. Third, the cross-sectional design of our study did not take into account the duration following diagnosis of the CMD. Lastly, no information was available regarding whether individuals with a CMD had received a nutritional counselling or particular recommendations by medical staff or other stakeholders following their diagnosis.

In conclusion, these results indicate that in adults with CMDs, a healthy diet continues to represent a public health challenge in terms of prevention of CMDs complications. Although some specific recommendations seem well integrated, continuing to encourage healthy habits regarding physical activity, tobacco smoking and alcohol consumption, intakes of fruit and legumes, whole grain products, and reduction of meat and processed meat rich in saturated fat and sodium intake, could improve its management after a CMD has been diagnosed. Although patients, who are at higher risk of developing a primary of secondary CVD (for example because they are more obese) could benefit more from following the recommendations, smoking status and alcohol consumption showed no difference between controls and patients. New strategies are needed to help patients adopt and maintain healthy dietary practices that will reduce their global risk of major cardiovascular events. In particular, following specific recommendations issued by international society could be a public health priority.

## Figures and Tables

**Table 1:** Comparisons of baseline characteristics among cases and controls for each cardiometabolic disorder, NutriNet-Santé Cohort, France, 2009–2016.

	Hypertension			Diabetes			Dyslipidaemia			Cardiovascular Diseases			Cardiometabolic Disorders		
	**Controls**	**Cases**	***p*** **^1^**	**Controls**	**Cases**	***p*** **^1^**	**Controls**	**Cases**	***p*** **^1^**	**Controls**	**Cases**	***p*** **^1^**	**Controls**	**Cases**	***p*** **^1^**
	***N*** **(%) or Mean ± SD**			***N*** **(%) or Mean ± SD**			***N*** **(%) or Mean ± SD**			***N*** **(%) or Mean ± SD**			***N*** **(%) or Mean ± SD**		
***N***	7801	7801		1759	1759		7063	7063		1098	1098		13285	13285	
**Sex**															
Men	2699 (34.6)	2699 (34.6)	1	751 (42.7)	751 (42.7)	1	2512 (35.3)	2512 (35.3)	1	560 (51.0)	560 (51.0)	1	4549 (32.9)	4549 (32.9)	1
Women	5102 (65.4)	5102 (65.4)		1008 (57.3)	1008 (57.3)		4600 (64.7)	4600 (64.7)		538 (49.0)	538 (49.0)		9293 (67.1)	9293 (67.1)	
**Age, years**	56.9 ± (7.73)	56.9 ± (7.73)	0.99	55.7 ± (8.61)	55.7 ± (8.61)	1	55.7 ± (8.55)	55.7 ± (8.55)	1	56.9 ± (7.75)	56.9 ± (8.17)	1	55.5 ± (8.39)	55.5 ± (8.41)	0.95
**Educational level, %**															
<2 years after high-school degree	3463 (45.9)	4338 (53.8)	<0.0001	771 (43.8)	987 (56.1)	<0.0001	3055 (43.2)	3175 (44.9)	0.22	505 (46.0)	543 (49.4)	0.1	5721 (43.1)	6421 (48.3)	<0.0001
≥2 years after high-school degree	4076 (54.1)	3725 (46.2)		998 (56.2)	772 (43.9)		3326 (56.8)	3888 (55.1)		593 (54.0)	555 (50.6)		7564 (56.9)	6864 (51.7)	
**Marital status, %**															
Single	529 (6.8)	603 (7.7)	0.054	133 (7.6)	182 (10.4)	0.004	528 (7.5)	619 (8.8)	0.006	81 (7.4)	77 (7.0)	0.22	966 (7.3)	1151 (8.7)	<0.0001
Divorced or widowed	1359 (17.4)	1309 (16.8)		278 (15.8)	304 (17.2)		1206 (17.1)	1124 (15.9)		165 (14.40)	195 (17.8)		2258 (17.0)	2155 (16.2)	
Married	5913 (75.8)	5889 (75.5)		1348 (76.6)	1273 (72.4)		5329 (75.5)	5320 (75.3)		852 (77.6)	826 (75.2)		10,061 (75.7)	9979 (75.1)	
**BMI categories, %**															
<25 kg/m^2^	5299 (67.9)	2917 (37.4)	<0.0001	1191 (67.1)	414 (23.1)	<0.0001	4798 (67.9)	3327 (47.1)	<0.0001	744 (67.8)	460 (41.9)	<0.0001	9050 (68.1)	5918 (44.6)	<0.0001
≥25 to <30 kg/m^2^	1963 (25.2)	2743 (35.2)		459 (26.1)	564 (32.1)		1760 (24.9)	2457 (34.8)		283 (25.8)	419 (38.2)		3315 (24.9)	4474 (33.7)	
≥30 kg/m^2^	539 (6.9)	2141 (27.4)		109 (6.2)	781 (44.4)		505 (7.1)	1279 (18.1)		71 (6.5)	219 (19.9)		920 (6.9)	2893 (21.8)	
**Number of 24 h-dietary records**	6.66 (3.02)	6.68 (3.00)	0.62	6.61 (3.03)	6.38 (3.04)	0.88	6.60 (3.02)	6.81 (3.01)	0.92	6.56 (3.01)	6.73 (3.06)	0.56	6.61 (3.03)	6.68 (3.01)	0.41
**Diet related to health conditions, %**	231 (1.48)	1204 (7.72)	<0.0001	55 (1.56)	805 (22.9)	<0.0001	202 (1.43)	1374 (9.73)	<0.0001	42 (1.91)	243 (11.1)	<0.0001	3.89 (1.46)	2272 (8.55)	<0.0001

^1^ *p* values are from the comparison between cases and controls using either MacNemar Chi square tests or paired Students *t* tests as appropriate. All statistical tests were 2-sided. Values are mean ± Standard deviation (SD) for continuous quantitative variables and frequencies (%) ± Standard deviation for quantitative categorical variables. BMI: Body Mass index (Weight (kg)/height (m)^2^).

**Table 2:** Percentage of participants ^2^ (%) complying ^3^ with the French recommendations in patients and their healthy controls, NutriNet-Santé Cohort, France, 2009–2016.

**Components of the French PNNS Recommendations**	**Hypertension**						**Diabetes**						**Dyslipidemia**					
	**Controls**		**Cases**				**Controls**		**Cases**				**Controls**		**Cases**			
	**%**	**95% CI**	**%**	**95% CI**	**RD**	***p*** **^1^**	**%**	**95% CI**	**%**	**95% CI**	**RD**	***p*** **^1^**	**%**	**95% CI**	**%**	**95% CI**	**RD**	***p*** **^1^**
Smoking status	11.5	10.7; 12.3	10.8	10.0; 11.5	−6.5	0.0757	11.7	10.1; 13.4	12.3	10.6; 14	4.9	0.6518	11.4	10.5; 12.2	12.0	11.2; 12.9	5.0	0.2417
Alcoholic beverages	89.2	88.4; 90.0	87.1	86.3; 87.9	−2.4	<0.0001	88.8	87.1; 90.4	87.0	85.3; 88.6	−2.1	0.1612	89.5	88.7; 90.3	87.7	86.9; 88.5	−2.1	0.0080
Physical activity	72.8	71.7; 73.9	66.3	65.2; 67.4	−9.8	**<0.0001**	73.3	71.0; 75.7	64.6	62.2; 66.9	−13.5	**<0.0001**	72.5	71.3; 73.7	67.1	65.9; 68.3	−8.0	**<0.0001**
Fruits and vegetables	59.6	58.4; 60.8	56.6	55.5; 57.8	−5.3	**0.0019**	60.9	58.5; 63.3	60.7	58.3; 63.2	−0.3	0.5821	58.8	57.6; 60.0	57.5	56.3; 58.7	−2.3	0.0831
Bread, Cereals, Legumes, potatoes	42.8	41.7; 43.9	44.9	43.8; 46.1	4.7	0.0173	44.8	42.4; 47.2	49.7	47.3; 52.1	9.9	**0.0048**	43.1	41.9; 44.3	46.0	44.8; 47.2	6.3	**0.0025**
Dairy products	32.2	31.0; 33.4	35.2	34.0; 36.4	8.5	**<0.0001**	32.7	30.3; 35.1	36.7	34.3; 39.1	10.9	0.0162	31.6	30.4; 32.8	32.6	31.4; 33.8	3.1	0.2698
Whole grains products	7.8	7.1; 8.4	5.9	5.2; 6.5	−32.2	**<0.0001**	6.8	5.5; 8.2	7.5	6.1; 8.9	9.3	0.5030	7.6	6.9; 8.2	6.5	5.8; 7.2	−16.9	**0.0714**
Meat, seafood and eggs	59.0	57.8; 60.2	60.4	59.2; 61.6	2.3	0.0569	57.9	55.4; 60.4	57.2	54.6; 59.7	−1.2	0.6154	59.1	57.9; 60.4	60.9	59.7; 62.2	3.0	0.0401
Seafood	53.5	52.2; 54.7	55.1	53.9; 56.4	2.9	0.0123	53.8	51.3; 56.3	53.5	50.9; 56.0	−0.6	0.9447	52.9	51.6; 54.2	58.0	56.7; 59.3	8.8	**<0.0001**
Added fat (butter, Oils, margarine)	81.3	80.3; 82.3	81.5	80.5; 82.5	0.2	0.6899	81.8	79.7; 83.9	77.6	75.4; 79.7	−5.4	0.0171	80.9	79.9; 81.9	82.8	81.8; 83.9	2.3	0.0791
Added simple sugars	82.4	81.5; 83.4	86.5	85.5; 87.4	4.7	<0.0001	80.4	78.7; 82.0	93.6	91.9; 95.3	14.1	**<0.0001**	81.7	80.7; 82.7	84.3	83.4; 85.3	3.1	0.0002
Non-alcoholic or sweetened beverages	58.0	56.8; 59.2	60.1	58.9; 61.3	3.5	0.0399	58.8	56.4; 61.2	64.3	61.8; 66.7	8.6	**0.0012**	58.8	57.5; 60.0	62.3	61.0; 63.5	5.6	**<0.0001**
Salt	43.5	42.5; 44.5	39.3	38.3; 40.3	−10.7	**<0.0001**	42.0	40.0; 44.1	29.7	27.6; 31.7	−41.4	**<0.0001**	43.1	42.1; 44.2	39.0	38.0; 40.1	−10.5	**0.0002**
PNNS-GS Score (4th Quartile)	21.1	20.5; 21.6	18.8	18.3; 19.3	−12.2	**0.0007**	19.4	18.4; 20.5	17.8	16.8; 18.8	−9.0	0.2297	20.2	19.7; 20.8	20.1	19.6; 20.7	−0.5	0.8818
PNNS-GS Score ^2,3^	9.4	9.38; 9.42	9.3	9.28; 9.33	−1.1	0.0023	9.3	9.25; 9.35	9.2	9.15; 9.25	−1.1	0.1667	9.4	9.38; 9.42	9.4	9.38; 9.42	0.0	0.4968
**Components of the French PNNS Recommendations**	**CVD**						**Cardiometabolic Disorders**											
	**Controls**		**Cases**				**Controls**		**Cases**									
	**%**	**95% CI**	**%**	**95% CI**	**RD**	***p*** **^1^**	**%**	**95% CI**	**%**	**95% CI**	**RD**	***p*** **^1^**						
Smoking status	11.2	9.1; 13.2	13.9	11.8; 16	19.4	0.0204	12.1	11.5; 12.8	12.1	11.5; 12.7	0.0	0.9255						
Alcoholic beverages	90.4	88.4; 92.3	88.5	86.6; 90.5	−2.1	0.1414	89.5	88.9; 90.1	88.1	87.5; 88.7	−1.6	<0.0001						
Physical activity	73.3	70.4; 76.2	65.8	62.9; 68.7	−11.4	0.0005	71.9	71.0; 72.7	66.3	65.4; 67.2	−8.4	<0.0001						
Fruits and vegetables	62.2	59.2; 65.2	57.7	54.6; 60.7	−7.8	0.0534	58.0	57.1; 58.9	56.1	55.2; 57	−3.4	0.0008						
Bread, Cereals, Legumes, potatoes	46.5	43.6; 49.5	47.4	44.5; 50.4	1.9	0.8691	42.9	42.0; 43.7	44.7	43.8; 45.5	4.0	0.0013						
Dairy products	32.9	29.9; 35.9	34.9	31.9; 37.9	5.7	0.2958	31.2	30.3; 32.1	32.8	31.9; 33.7	4.9	0.0121						
Whole grains products	6.6	4.9; 8.2	7.1	5.4; 8.7	7.0	0.5466	7.7	7.2; 8.2	6.4	5.9; 6.9	−20.3	<0.0001						
Meat, seafood and eggs	57.9	54.8; 61	60.3	57.2; 63.5	4.0	0.3766	58.3	57.4; 59.2	60.4	59.4; 61.3	3.5	0.0004						
Seafood	54.2	51.1; 57.3	55.7	52.6; 58.8	2.7	0.4518	52.5	51.5; 53.4	55.6	54.6; 56.5	5.6	<0.0001						
Added fat (butter, Oils, margarine)	81.4	78.9; 83.9	81.6	79.1; 84.1	0.2	0.8181	82.0	81.2; 82.8	82.2	81.4; 82.9	0.2	0.5562						
Added simple sugars	81.8	79.4; 84.2	83.6	81.2; 86	2.2	0.4031	81.8	81; 82.5	84.9	84.1; 85.6	3.7	<0.0001						
Non-alcoholic or sweetened beverages	58.3	55.2; 61.4	60.7	57.7; 63.8	4.0	0.3431	58.8	57.9; 59.7	61.0	60.1; 61.9	3.6	0.0003						
Salt	42.6	40; 45.2	37.6	35; 40.3	−13.3	0.0134	43.8	43; 44.6	39.9	39.1; 40.7	−9.8	<0.0001						
PNNS-GS Score (4th Quartile)	19.5	18.1; 20.8	17.0	15.7; 18.2	−14.7	0.1363	20.0	19.6; 20.4	19.3	18.9; 19.7	−3.5	0.1312						
PNNS-GS Score 2,3	9.4	9.34; 9.46	9.3	9.24 ; 9.36	−1.1	0.2174	9.4	9.38; 9.42	9.3	9.28; 9.32	−1.1	0.0118						

^1^ *p* values are from the comparison between cases and controls using multivariable mixed logistic regression. Bold *p*-values are < 0.01 with a relative difference above 5%. Models were adjusted for age, sex, energy intake, number of dietary records and season of dietary assessment (spring/summer or autumn/winter). RD: Relative difference in proportion between cases and controls. A positive relative difference shows higher intake of nutrients or foods groups in cases and a negative relative difference shows lower intakes in controls. SEM: Standard error of the mean, CVD: Cardiovascular diseases, CI: Confidence Intervals. ^2^ values are means for the estimates and 95% CI. ^3^: See Tables S1 and S2 for details on construction of the PNNS-GS score or the threshold which defined each recommendation.

**Table 3:** Multivariable ^1^ comparisons of food groups intakes among patients and their healthy controls, NutriNet-Santé Cohort, France, 2009–2016.

**Foods Groups Intakes (g/day)**	**Hypertension**						**Diabetes**						**Dyslipidemia**					
	**Controls**		**Cases**				**Controls**		**Cases**				**Controls**		**Cases**			
	**Mean**	**SEM**	**Mean**	**SEM**	**RD**	***p*** **^2^**	**Mean**	**SEM**	**Mean**	**SEM**	**RD**	***p*** **^2^**	**Mean**	**SEM**	**Mean**	**SEM**	**RD**	***p*** **^2^**
*Alcoholic beverages*	129.3	1.91	133.2	1.91	3.0	0.1142	131.9	4.25	124.4	4.32	−6.0	0.1904	127.3	1.99	132.1	1.98	3.6	0.0669
*Wine*	90.1	1.43	95.2	1.42	5.4	0.007	91.5	2.98	86.3	3.03	−6.0	0.1993	87.8	1.46	93.2	1.45	5.8	0.005
*Fruits*	291.4	2.02	273.6	2.02	−6.5	<0.0001	302.3	4.26	262.5	4.33	−15.2	<0.0001	290.0	2.1	272.7	2.09	−6.3	<0.0001
*Vegetables*	240.8	1.43	238.2	1.43	−1.1	0.1666	239.8	3.22	259.2	3.28	7.5	<0.0001	238.9	1.48	237.4	1.46	−0.6	0.4458
*Starchy foods (pasta. rice. semolina. bread. flour. others cereals)*	191.5	1.05	201.3	1.05	4.9	<0.0001	198.0	2.24	206.0	2.28	3.9	0.0083	193.0	1.11	202.0	1.1	4.5	<0.0001
*Whole grain products*	41.1	0.60	35.9	0.60	−14.4	<0.0001	41.0	1.30	42.8	1.32	4.1	0.3185	40.7	0.63	38.0	0.63	−7.1	0.0012
*Pulses*	13.2	0.26	11.7	0.26	−12.8	<0.0001	12.8	0.66	13.9	0.67	8.1	0.2041	12.9	0.28	12.3	0.28	−4.9	0.1304
*Red meat and offal*	46.9	0.48	55.6	0.48	15.6	<0.0001	45.4	1.02	60.7	1.03	25.2	<0.0001	47.1	0.5	53.5	0.49	12.1	<0.0001
*Processed meat*	29.3	0.34	35.0	0.34	16.2	<0.0001	30.0	0.76	39.3	0.78	23.7	<0.0001	29.8	0.36	33.3	0.36	10.5	<0.0001
*Poultry*	25.6	0.36	28.8	0.36	11.1	<0.0001	25.5	0.8	32.4	0.82	21.4	<0.0001	25.5	0.38	28.4	0.38	10.3	<0.0001
*Fish and seafood*	47.3	0.52	49.7	0.52	4.9	0.0004	49.8	1.13	50.2	1.15	0.8	0.8028	47.0	0.54	50.9	0.54	7.5	<0.0001
*Eggs*	15.1	0.23	14.7	0.23	−2.7	0.3047	15.7	0.51	15.7	0.51	0.3	0.9463	15.0	0.23	12.6	0.23	−19.0	<0.0001
*Cheese*	37.7	0.31	37.7	0.31	0.1	0.9400	39.3	0.67	40.0	0.68	1.9	0.3925	37.8	0.33	36.7	0.32	−2.9	0.0141
*Dairy products (milk. yoghurt)*	152.9	1.83	162.7	1.82	6.0	<0.0001	156.2	3.89	183.4	3.95	14.8	<0.0001	153.9	1.91	160.3	1.9	4.0	0.0114
*Added oils and margarine*	20.1	0.14	20.9	0.14	3.8	<0.0001	20.0	0.3	22.7	0.3	12.0	<0.0001	20.3	0.15	22.2	0.15	8.4	<0.0001
*Butter and others animal added fat*	14.6	0.15	14.8	0.15	1.3	0.3267	15.3	0.32	16.0	0.32	4.4	0.085	14.8	0.15	13.3	0.15	−11.4	<0.0001
*Fatty and sweet pastries*	59.1	0.55	56.2	0.55	−5.2	<0.0001	60.6	1.15	52.4	1.17	−15.6	<0.0001	60.9	0.58	58.8	0.58	−3.5	0.0069
*Fatty and sweet products (without feculents)*	15.3	0.29	15.3	0.29	−0.1	0.9755	16.4	0.65	14.9	0.66	−10.1	0.0832	15.4	0.32	15.9	0.32	3.1	0.2398
*Sweet products*	55.3	0.62	50.6	0.61	−9.3	<0.0001	57.8	1.24	37.6	1.26	−53.8	<0.0001	56.2	0.65	52.3	0.64	−7.4	<0.0001
*Sweetened drinks (non-alcoholic)*	22.4	0.83	25.7	0.83	12.8	0.003	22.7	2.14	20.7	2.17	−9.6	0.4904	24.3	0.88	24.9	0.87	2.2	0.6285
*Tea*	186.6	3.30	146.9	3.30	−27.0	<0.0001	187.7	6.54	129.0	6.65	−45.5	<0.0001	184.7	3.59	164.0	3.56	−12.6	<0.0001
*Coffee*	180.4	2.37	193.0	2.36	6.5	<0.0001	173.4	4.97	211.5	5.05	18.0	<0.0001	182.4	2.55	196.0	2.53	6.9	<0.0001
**Foods Groups Intakes (g/day)**	**CVD**						**Cardiometabolic Disorders**											
	**Controls**		**Cases**				**Controls**		**Cases**									
	**Mean**	**SEM**	**Mean**	**SEM**	**RD**	***p*** **^2^**	**Mean**	**SEM**	**Mean**	**SEM**	**RD**	***p*** **^2^**						
*Alcoholic beverages*	129.9	4.78	129.0	4.81	−0.7	0.8911	125.7	1.44	128.4	1.44	2.1	0.1573						
*Wine*	89.3	3.48	91.2	3.5	2.1	0.6937	86.9	1.07	90.1	1.07	3.6	0.0203						
*Fruits*	301.8	5.49	274.0	5.52	−10.1	0.0002	287.6	1.55	272.7	1.54	−5.5	<0.0001						
*Vegetables*	240.6	3.81	240.2	3.83	−0.2	0.9387	238.5	1.11	236.8	1.1	−0.7	0.2391						
*Starchy foods (pasta. rice. semolina. bread. flour. others cereals)*	199.2	2.87	208.2	2.89	4.4	0.0211	191.9	0.81	200.2	0.81	4.1	<0.0001						
*Whole grain products*	40.5	1.64	40.9	1.65	1.0	0.8614	41.4	0.47	37.4	0.47	−10.6	<0.0001						
*Pulses*	12.9	0.73	12.8	0.74	−0.8	0.8872	13.3	0.21	12.2	0.21	−9.0	<0.0001						
*Red meat and offal*	46.3	1.23	52.2	1.24	11.3	0.008	46.9	0.37	53.8	0.37	12.7	<0.0001						
*Processed meat*	28.9	0.87	31.4	0.87	7.9	0.009	29.4	0.26	33.7	0.26	12.8	<0.0001						
*Poultry*	27.2	0.36	28.7	0.38	5.0	0.008	25.5	0.28	28.5	0.28	10.5	<0.0001						
*Fish and seafood*	49.7	1.39	50.7	1.4	2.0	0.5797	46.9	0.4	49.5	0.4	5.3	<0.0001						
*Eggs*	15.7	0.6	13.6	0.6	−15.4	0.01	14.9	0.18	13.8	0.18	−7.9	<0.0001						
*Cheese*	39.8	0.87	37.9	0.87	−5.0	0.1255	37.4	0.24	36.9	0.24	−1.4	0.0987						
*Dairy products (milk. yoghurt)*	153.8	4.93	166.5	4.96	7.6	0.0605	153.2	1.42	161.9	1.42	5.3	<0.0001						
*Added oils and margarine*	19.8	0.38	22.2	0.38	10.8	<0.0001	19.9	0.11	21.3	0.11	6.6	<0.0001						
*Butter and others animal added fat*	15.6	0.39	14.6	0.4	−6.8	0.0681	14.5	0.11	14.0	0.11	−3.6	0.0013						
*Fatty and sweet pastries*	58.4	1.45	58.4	1.45	−0.1	0.9887	60.3	0.43	58.0	0.43	−3.9	0.0001						
*Fatty and sweet products (without feculents)*	15.8	0.71	15.5	0.72	−1.9	0.7391	15.5	0.23	15.7	0.23	1.3	0.3632						
*Sweet products*	58.7	1.66	52.2	1.67	−12.4	0.0045	55.8	0.48	51.2	0.48	−9.0	<0.0001						
*Sweetened drinks (non-alcoholic)*	24.0	2.42	24.6	2.43	2.4	0.8559	23.9	0.67	25.6	0.67	6.6	0.0429						
*Tea*	184.6	8.49	157.4	8.54	−17.3	0.0095	187.3	2.64	155.5	2.64	−20.5	<0.0001						
*Coffee*	173.8	6.13	186.5	6.16	6.8	0.1321	182.4	1.88	193.7	1.87	5.8	<0.0001						

^1^ Models were adjusted for age, sex, energy intake, number of dietary records and season of dietary assessment (spring/summer or autumn/winter). ^2^ *p* values are from the comparison between cases and controls using multivariable mixed linear regressions (taking into account the matching). Bold *p*-values are < 0.01 with a relative difference above 5%. RD: Relative difference in intakes between cases and controls. A positive relative difference shows higher intakes of nutrient in cases and a negative relative difference shows lower intakes in controls. SEM: Standard error of the mean, CVD: Cardiovascular diseases.

**Table 4:** Multivariable ^1^ comparisons of nutrient intakes in patients and their healthy controls, NutriNet-Santé Cohort, France, 2009–2016.

**Nutrients Intakes**	**Hypertension**						**Diabetes**						**Dyslipidemia**					
	**Controls**		**Cases**				**Controls**		**Cases**				**Controls**		**Cases**			
	**Mean**	**SEM**	**Mean**	**SEM**	**RD**	***p*** **^2^**	**Mean**	**SEM**	**Mean**	**SEM**	**RD**	***p*** **^2^**	**Mean**	**SEM**	**Mean**	**SEM**	**RD**	***p*** **^2^**
*Energy (kcal/day)*	1985.2	5.49	1950.3	5.52	−1.8	<0.0001	1991.9	12.23	1939.4	12.48	−2.7	0.0016	1993.2	5.76	1948.3	5.76	−2.3	<0.0001
*Proteins (g/day)*	80.3	0.18	84.3	0.18	4.8	<0.0001	81.6	0.39	89.6	0.4	9.0	**<0.0001**	80.5	0.19	83.8	0.19	4	<0.0001
*Lipids (g/day)*	78.9	0.15	78.7	0.15	−0.3	0.1986	80.2	0.32	83.4	0.33	3.8	<0.0001	79.7	0.16	78.6	0.16	−1.3	<0.0001
*Saturated fatty acids (g/day)*	31.7	0.09	31.8	0.09	0.3	0.4972	32.4	0.18	33.5	0.19	3.4	<0.0001	32.1	0.09	31.1	0.09	−3.1	<0.0001
*Monounsaturated fatty acids (g/day)*	29.7	0.08	29.4	0.08	−1.1	0.0025	30.1	0.17	31.1	0.17	3.4	<0.0001	29.9	0.09	29.6	0.09	−1	0.0081
*Polyunsaturated fatty acids (g/day)*	11.6	0.05	11.5	0.05	−0.8	0.1477	11.7	0.11	12.4	0.11	5.6	**<0.0001**	11.6	0.05	11.9	0.05	2	0.0009
*Added fats (g/day)*	23.6	0.13	23.8	0.13	0.7	0.3399	24.0	0.28	25.7	0.29	6.6	**<0.0001**	23.8	0.14	23.4	0.14	−2	0.0099
*Added plant fats (g/day)*	15.2	0.12	15.4	0.12	1.2	0.2457	15.3	0.24	16.5	0.25	7.4	**0.0002**	15.3	0.12	16.0	0.12	4.2	<0.0001
*Added animal fats (g/day)*	8.4	0.09	8.3	0.09	−0.1	0.9366	8.7	0.19	9.2	0.19	5.2	0.0602	8.6	0.09	7.2	0.09	−16.0	**<0.0001**
*Cholesterol (mg/day)*	316.7	1.38	330.9	1.38	4.3	<0.0001	325.4	2.97	347.8	3.02	6.5	**<0.0001**	318.8	1.42	314.1	1.4	−1.5	0.0131
*Total carbohydrates (g/day)*	193.1	0.41	188.5	0.41	−2.4	<0.0001	198.6	0.86	183.8	0.88	−8.1	**<0.0001**	194.7	0.44	192.5	0.44	−1.1	0.0002
*Complex carbohydrates (g/day)*	89.1	0.31	83.7	0.31	−6.5	**<0.0001**	91.9	0.65	75.8	0.67	−21.0	**<0.0001**	89.9	0.33	85.8	0.33	−4.8	<0.0001
*Sugars (g/day)*	103.5	0.32	104.3	0.32	0.8	0.0608	106.2	0.69	107.4	0.7	1.2	0.1739	104.3	0.34	106.3	0.34	1.8	<0.0001
*Included added simple sugars (g/day)*	32.5	0.21	29.3	0.21	−11.0	**<0.0001**	33.7	0.45	21.9	0.45	−54.0	**<0.0001**	33.3	0.23	30.8	0.23	−8	**<0.0001**
*Fibre (g/day)*	21.0	0.08	20.0	0.07	−4.7	<0.0001	21.3	0.16	21.0	0.16	−1.4	0.1635	20.9	0.08	20.5	0.08	−2	0.0001
*Alcohol (g/day)*	11.0	0.16	11.6	0.16	5.6	0.0018	11.1	0.34	10.9	0.35	−2.0	0.6369	10.8	0.16	11.4	0.16	5.9	**0.0015**
*Calcium (mg/day)*	912.6	3.02	919.7	3.02	0.8	0.0742	934.9	6.52	970.6	6.63	3.7	0.0001	917.0	3.14	919.5	3.11	0.3	0.5464
*Potassium (mg/day)*	3106.0	7.86	3107.3	7.84	0.0	0.8962	3154.6	17.05	3251.9	17.33	3.0	<0.0001	3105.1	8.16	3134.4	8.1	0.9	0.0064
*B-carotène (ug/day)*	3693.0	28.8	3558.1	28.8	−3.8	0.0004	3750.5	63.17	3790.5	64.21	1.1	0.6387	3687.4	30.6	3605.3	30.37	−2.3	0.0416
*Vitamin B12 (mg/day)*	5.8	0.06	6.2	0.06	5.8	**<0.0001**	6.1	0.13	6.3	0.13	4.4	0.1121	5.8	0.06	6.1	0.06	4.3	0.0016
*Vitamin C (mg/day)*	117.2	0.75	113.7	0.75	−3.0	0.0005	118.5	1.5	113.5	1.53	−4.4	0.0136	116.6	0.78	113.9	0.77	−2.4	0.0078
*Vitamin D (µg/day)*	2.9	0.03	2.9	0.03	1.4	0.1791	2.9	0.05	3.0	0.05	2.7	0.2562	2.9	0.03	2.9	0.03	2.7	0.0173
*Salt (g/day)*	8.8	0.03	9.2	0.03	4.3	<0.0001	8.9	0.06	9.9	0.06	10.0	**<0.0001**	8.8	0.03	9.1	0.03	3.5	<0.0001
*Sodium (mg/day)*	2764.4	8.41	2888.9	8.4	4.3	<0.0001	2813.6	18.64	3127.1	18.95	10.0	**<0.0001**	2775.9	8.74	2876.8	8.68	3.5	<0.0001
**Nutrients Intakes**	**CVD**						**Cardiometabolic Disorders**											
	**Controls**		**Cases**				**Controls**		**Cases**									
	**Mean**	**SEM**	**Mean**	**SEM**	**RD**	***p*** **^2^**	**Mean**	**SEM**	**Mean**	**SEM**	**RD**	***p*** **^2^**						
*Energy (kcal/day)*	1985.9	15.46	1871.1	15.55	−6.1	**<0.0001**	1989.1	4.24	1954.0	4.25	−1.8	<0.0001						
*Proteins (g/day)*	82.3	0.48	84.5	0.48	2.7	0.0005	80.2	0.14	83.5	0.14	3.9	<0.0001						
*Lipids (g/day)*	80.5	0.41	79.9	0.41	−0.8	0.2813	78.6	0.12	78.4	0.12	−0.2	0.2390						
*Saturated fatty acids (g/day)*	32.6	0.24	32.0	0.24	−1.8	0.0791	31.6	0.07	31.4	0.07	−0.8	0.0042						
*Monounsaturated fatty acids (g/day)*	30.1	0.21	29.9	0.22	−0.7	0.4625	29.5	0.06	29.4	0.06	−0.4	0.1725						
*Polyunsaturated fatty acids (g/day)*	11.7	0.13	12.0	0.14	2	0.1966	11.5	0.04	11.6	0.04	1.1	0.0125						
*Added fats (g/day)*	24.2	0.36	24.3	0.36	0.7	0.7516	23.3	0.1	23.4	0.1	0.4	0.5206						
*Added plant fats (g/day)*	15.2	0.31	16.1	0.31	5.9	0.0254	15.0	0.09	15.5	0.09	3.5	<0.0001						
*Added animal fats (g/day)*	9.0	0.23	8.2	0.24	−9.6	0.0137	8.3	0.07	7.9	0.07	−5.9	**<0.0001**						
*Cholesterol (mg/day)*	325.9	3.75	323.1	3.77	−0.9	0.5845	315.1	1.06	321.1	1.06	1.9	<0.0001						
*Total carbohydrates (g/day)*	198.3	1.11	196.7	1.11	−0.8	0.2904	193.5	0.32	189.9	0.32	−1.9	<0.0001						
*Complex carbohydrates (g/day)*	91.5	0.85	86.5	0.86	−5.7	**<0.0001**	89.0	0.24	84.5	0.24	−5.3	**<0.0001**						
*Sugars (g/day)*	106.3	0.9	109.6	0.91	3.0	0.0071	104.0	0.25	104.9	0.25	0.8	0.0090						
*Included added simple sugars (g/day)*	33.5	0.59	31.3	0.59	−7.0	**0.0067**	32.8	0.17	30.1	0.17	−9.2	**<0.0001**						
*Fibre (g/day)*	21.4	0.21	21.0	0.21	−1.8	0.1925	20.8	0.06	20.2	0.06	−3.1	<0.0001						
*Alcohol (g/day)*	10.8	0.41	11.3	0.41	3.8	0.4548	10.7	0.12	11.1	0.12	4.1	0.0043						
*Calcium (mg/day)*	936.7	8.06	934.4	8.1	−0.2	0.8376	912.8	2.33	916.6	2.33	0.4	0.2112						
*Potassium (mg/day)*	3156.5	21.11	3148.6	21.23	−0.3	0.7855	3085.2	6.04	3103.2	6.02	0.6	0.0222						
*B-carotène (ug/day)*	3690.8	77.72	3540.3	78.16	−4.3	0.1581	3680.8	22.53	3560.4	22.5	−3.4	<0.0001						
*Vitamin B12 (mg/day)*	6.0	0.17	6.0	0.17	0.2	0.9440	5.8	0.05	6.0	0.05	4.3	<0.0001						
*Vitamin C (mg/day)*	118.2	1.99	113.5	2.01	−4.2	0.0847	115.8	0.58	113.1	0.58	−2.4	0.0004						
*Vitamin D (µg/day)*	3.0	0.07	3.0	0.07	0.7	0.8101	2.8	0.02	2.9	0.02	2.1	0.0071						
*Salt (g/day)*	9.0	0.07	9.4	0.07	4.6	<0.0001	8.8	0.02	9.1	0.02	3.6	<0.0001						
*Sodium (mg/day)*	2824.3	22.91	2960.1	23.04	4.6	<0.0001	2758.8	6.4	2863.2	6.38	3.6	<0.0001						

^1^ Models were adjusted for age, sex, energy intake, number of dietary records and season of dietary assessment (spring/summer or autumn/winter). ^2^ *p* values are from the comparison between cases and controls using multivariable mixed linear regressions (taking into account the matching). Bold *p*-values are < 0.01 with a relative difference above 5%. RD: Relative difference in intakes between cases and controls. A positive relative difference shows higher intake of nutrients in cases and a negative relative difference shows lower intakes in controls. SEM: Standard error of the mean, CVD: Cardiovascular diseases.

**Table 5:** Multivariable ^1^ comparisons of intakes of major classes of polyphenols in patients and their healthy controls, NutriNet-Santé Cohort, France, 2009–2016.

**Polyphenol Intakes (mg/day)**	**hypertension**						**Diabetes**						**Dyslipidemia**					
	**Controls**		**Cases**				**Controls**		**Cases**				**Controls**		**Cases**			
	**Mean**	**SEM**	**Mean**	**SEM**	**RD**	***p*** **^2^**	**Mean**	**SEM**	**Mean**	**SEM**	**RD**	***p*** **^2^**	**Mean**	**SEM**	**Mean**	**SEM**	**RD**	***p*** **^2^**
*Anthocyanins*	54.28	0.93	50.18	0.93	−8.2	**0.0008**	58.36	1.93	43.80	1.96	−33.2	**<0.0001**	52.69	0.91	49.97	0.9	−5.4	0.0231
*Dihydrochalcones*	3.44	0.05	3.24	0.05	−6.2	0.0016	3.56	0.11	3.39	0.11	−5.0	0.2335	3.40	0.05	3.34	0.05	−1.8	0.3637
*Dihydroflavonols*	3.47	0.06	3.45	0.06	−0.6	0.8317	3.53	0.12	3.09	0.12	−14.2	**0.0061**	3.36	0.06	3.49	0.06	3.7	0.0968
*Catechins*	132.74	1.78	107.1	1.78	−23.9	**<0.0001**	133.89	3.56	96.16	3.62	−39.2	**<0.0001**	131.24	1.94	117.79	1.92	−11.4	**<0.0001**
*Theaflavins*	19.15	0.38	15.62	0.38	−22.6	**<0.0001**	19.25	0.76	13.51	0.77	−42.5	**<0.0001**	19,71	0,42	17,46	0,42	−13.0	**<0.0001**
*Flavanons*	28.51	0.40	28.14	0.39	−1.3	0.4730	29.46	0.78	23.42	0.79	−25.8	**<0.0001**	28.97	0.41	27.80	0.40	−4.2	0.0293
*Flavones*	26.63	0.18	26.75	0.18	0.4	0.6100	27.31	0.38	27.04	0.39	−1.0	0.6037	26.92	0.19	27.17	0.19	0.9	0.3150
*Flavonols*	73.50	0.55	69.59	0.55	−5.6	**<0.0001**	73.32	1.16	70.28	1.18	−4.3	0.0532	72.55	0.58	72.14	0.57	−0.6	0.5885
*Hydroxybenzoic acids*	64.80	1.01	54.03	1.01	−19.9	**<0.0001**	64.26	2.01	51.34	2.05	−25.2	**<0.0001**	63.20	1.07	58.88	1.06	−7.3	**0.0022**
*Hydroxycinnamic acids*	618.75	5.36	649.10	5.35	4.7	<0.0001	611.10	11.25	695.22	11.44	12.1	**<0.0001**	620.84	5.75	657.39	5.71	5.6	**<0.0001**
*Total polyphenols*	1138.09	5.78	1107.04	5.77	−2.8	<0.0001	1138.91	12.19	1123.9	12.39	−1.3	0.3622	1133.45	6.23	1141.27	6.18	0.7	0.3405
**Polyphenol Intakes (mg/day)**	**CVD**						**Cardiometabolic Disorders**											
	**Controls**		**Cases**				**Controls**		**Cases**									
	**Mean**	**SEM**	**Mean**	**SEM**	**RD**	***p*** **^2^**	**Mean**	**SEM**	**Mean**	**SEM**	**RD**	***p*** **^2^**						
*Anthocyanins*	59.53	2.48	50.16	2.49	−18.7	**0.0059**	52.13	0.69	48.33	0.69	−7.9	**<0.0001**						
*Dihydrochalcones*	3.50	0.13	3.33	0.13	−5.1	0.3442	3.41	0.04	3.28	0.04	−4.0	0.0045						
*Dihydroflavonols*	3.50	0.14	3.41	0.15	−2.6	0.6808	3.29	0.04	3.31	0.04	0.6	0.6902						
*Catechins*	130.95	4.65	114.29	4.68	−14.6	**0.0091**	132.44	1.43	112.25	1.42	−18.0	**<0.0001**						
*Theaflavins*	18.69	0.96	15.99	0.97	−16.9	**0.0041**	19.28	0.30	16.49	0.3	−16.9	**<0.0001**						
*Flavanons*	29.79	1.02	27.16	1.03	−9.7	0.0614	28.56	0.30	27.72	0.3	−3.0	0.0322						
*Flavones*	27.45	0.46	27.35	0.46	−0.4	0.8735	26.67	0.14	26.74	0.14	0.3	0.6714						
*Flavonols*	73.14	1.44	71.23	1.45	−2.7	0.3360	73.17	0.43	70.12	0.43	−4.3	<0.0001						
*Hydroxybenzoic acids*	65.21	2.48	59.02	2.49	−10.5	0.0690	64.3	0.80	56.87	0.80	−13.1	**<0.0001**						
*Hydroxycinnamic acids*	620.53	14.24	658.11	14.32	5.7	0.0545	618.69	4.24	649.36	4.23	4.7	<0.0001						
*Total polyphenols*	1145.35	15.57	1133.52	15.66	−1.0	0.5796	1133.02	4.60	1116.02	4.58	−1.5	0.0044						

^1^ Models were adjusted for age, sex, energy intake, number of dietary records and season of dietary assessment (spring/summer or autumn/winter). ^2^ *p* values are from the comparison between cases and controls using multivariable mixed linear regressions (taking into account the matching). Bold *p*-values are <0.01 with a relative difference above 5%. RD: Relative difference in intakes between cases and controls. A positive relative difference shows higher intake of nutrient in cases and a negative relative difference shows lower intakes in controls. SEM: Standard error of the mean. CVD: Cardiovascular diseases.

## Supplementary Materials

The following are available online at www.mdpi.com/2072-6643/9/6/546/s1, Table S1: PNNS-GS: components and scores according to PNNS recommendations, Table S2: Definition of portion sizes corresponding to one serving of each food group, Table S3: Percentage of participants (%) complying with the specific recommendations in patients and their healthy controls, NutriNet-Santé Cohort, France, 2009–2016, Table S4: Multivariable1 comparisons of macronutrient intakes in patients and their healthy controls, expressed as percentage to total energy, NutriNet-Santé Cohort, France, 2009–2016.
