# Supplementary material for: Compliance with Nutritional and Lifestyle Recommendations in 13,000 Patients with a Cardiometabolic Disease from the Nutrinet-Santé Study
Source: Nutrients. 2017 May 26;9(6):546. doi: 10.3390/nu9060546 (PMC5490525; doi:10.3390/nu9060546)
Supplement: Supplementary file 1 [file nutrients-09-00546-s001.docx]

**Table S1.** PNNS–GS: components and scores according to PNNS recommendations.

|  | **Recommendation** | **Scoring criteria ^1^** | **Score** |
| --- | --- | --- | --- |
| 1.Fruits and vegetables | At least 5/d | [0–3.5] | 0 |
|  |  | [3.5–5] | 0.5 |
|  |  | [5–7.5] | 1 |
|  |  | ≥7.5 | 2 |
| 2.Bread, cereals, potatoes and legumes | At each meal according to appetite | [0–1] | 0 |
|  |  | [1–3] | 0.5 |
|  |  | [3–6] | 1 |
|  |  | ≥6 | 0.5 |
| 3.Whole grain food | Choose whole grains and whole–grain breads more often | [0–1/3] | 0 |
|  |  | [1/3–2/3] | 0.5 |
|  |  | ≥2/3 | 1 |
| 4.Milk and dairy products | 3/d (≥55–years–old: 3 to 4/d) | [0–1] | 0 |
|  |  | [1–2.5] | 0.5 |
|  |  | [2.5–3.5] (55–years–old: [2.5–4.5]) | 1 |
|  |  | >3.5 (55–years–old : >4.5) | 0 |
| 5.Meat, poultry seafood and eggs | 1 to 2/d | 0 | 0 |
|  |  | [0–1] | 0.5 |
|  |  | [1–2] | 1 |
|  |  | >2 | 0.5 |
| 6.Seafood | At least 2/week | < 2/week | 0 |
|  |  | ≥ 2/week | 1 |
| 7.Added fat | Limit consumption | Lipids from added fat >16% EI ^3^/d | 0 |
|  |  | Lipids from added fat ≤16% EI/d | 1 |
| 8.Vegetable added fat | Favor fat of vegetable origin | No use of vegetable oil or ratio vegetable oil/total added fats≤0.5 | 0 |
|  |  | No use of added fats or ratio vegetable oil/total added fats >0.5 | 1 |

| 9.Sweetened foods | Limit consumption | Added sugar from sweetened foods ≥17.5% EI/d | −0.5 |
| --- | --- | --- | --- |
|  |  | Added sugar from sweetened foods 17.5–12.5% EI/d | 0 |
|  |  | Added sugar from sweetened foods <12.5% EI/d | 1 |
| Beverages |  |  |  |
| 10.Non–alcoholic beverages | Drink water as desired  Limit sweetened beverages: no more than 1 glass/d | <1l water and >250 ml soda/d | 0 |
|  |  | ≥1l water and >250 ml soda/d | 0.5 |
|  |  | <1l water and ≤250 ml soda/d | 0.75 |
|  |  | ≥1l water and ≤250 ml soda/d | 1 |
| 11.Alcohol | Women advised to drink ≤2 glasses of wine/d and ≤3 glasses/d for men | Ethanol >20 g/d for women and >30g/d for men | 0 |
|  |  | Ethanol ≤20 g/d for women and ≤30g/d for men | 0.8 |
|  |  | Abstainers and irregular consumers (<once a week) | 1 |
| 12.Salt 2 | Limit consumption | >12g/d | –0.5 |
|  |  | [10–12] g/d | 0 |
|  |  | [8–10] g/d | 0.5 |
|  |  | [6–8] g/d | 1 |
|  |  | ≤6 g/d | 1.5 |
| 13.Physical activity | At least the equivalent of 30 min/d of brisk walking | [0–30[ min/d | 0 |
|  |  | [30–60[ min/d | 1 |
|  |  | ≥60 min/d | 1.5 |
| Penalty on energy intake |  | EI/EE≤1.05 | 0 |
| Ex: EI/EE=1.10 🡪 Total score reduced by 10%. |  | EI/EE>1.05 🡪 X=EI/EE–1 | Total score –X% |

^1^ Servings per day unless otherwise indicated ^2^ Established according to French recommended dietary allowances ^3^ EI: energy intake without alcohol

**Table S2.** Definition of portion sizes corresponding to one serving of each food group.

| **Foods groups** | **Serving** |
| --- | --- |
| Fruit | 80 g |
| Vegetables | 80 g |
| Pure fruit juices | 150 mL |
| Vegetables juices | 150 mL |
| Potatoes | 150 g |
| Other starches and tubers | 150 g |
| Bread, rusks (+ wholegrain related products) | 50 g |
| Breakfast cereals (+ wholegrain related products) | 30 g |
| Flours (+wholegrain flours) | 30 g |
| Pasta (+wholegrain pasta) | 200 g |
| Rice (+wholegrain rice) | 200 g |
| Semolina | 200 g |
| Other cereals (+wholegrain cereals) | 200 g |
| Pulses | 200 g |
| Fish | 100 g |
| Seafood | 100 g |
| Meat | 100 g |
| Poultry | 100 g |
| Pork hams and poultry cuts | 100 g |
| Offal | 100 g |
| Eggs | 2 eggs = 100g |
| Milk | 150 mL |
| Cheese | 30 g |
| Yoghurts | 125 g |
| Fromages blancs | 100 g |
| « Petits suisses » | 120 g |
| Sweetened beverages | 250 mL |
| Unsweetened beverages | 250 mL |

**Table S3.** Table S3: Percentage of participants (%) complying with the specific recommendations in patients and their healthy controls, NutriNet-Santé Cohort, France, 2009–2016.

| **Components of specific recommendation** | **Hypertension** | | | | | | **Diabetes** | | | | | | **Dyslipidemia** | | | | | |
| --- | --- | --- | --- | --- | --- | --- | --- | --- | --- | --- | --- | --- | --- | --- | --- | --- | --- | --- |
| **recommendations** | **controls** | | **cases** | |  |  | **controls** | | **cases** | |  |  | **controls** | | **cases** | |  |  |
|  | **%** | **95% CI** | **%** | **95% CI** | **RD** | ***P* ^1^** | **%** | **95% CI** | **%** | **95% CI** | **RD** | ***P* ^1^** | **%** | **95% CI** | **%** | **95% CI** | **RD** | ***P* ^1^** |
| Sodium <2400 mg | 37,6 | 36.6 ; 38.6 | 33,9 | 32.9 ; 34.9 | –10,9 | **0.0001** | 35,6 | 33.6 ; 37.7 | 25,1 | 23.1 ; 27.2 | –41,8 | **<0.0001** | 37,1 | 36.1 ; 38.2 | 33,6 | 32.6 ; 34.7 | –10,4 | **0.0049** |
| Saturated fat ≥ 10% of energy intake | 8,3 | 7.7 ; 8.9 | 7,2 | 6.6 ; 7.8 | –15,3 | **0.0048** | 7,9 | 6.6 ; 9.1 | 5,1 | 3.9 ; 6.4 | –54,9 | **0.0002** | 7,9 | 7.2 ; 8.6 | 9,0 | 8.3 ; 9.6 | 12,2 | 0.0529 |
| Fibers ≥ 30 g/day | 10,8 | 10.2 ; 11.5 | 7,7 | 7.1 ; 8.4 | –40,3 | **<0.0001** | 11,3 | 9.8 ; 12.8 | 9,1 | 7.7 ; 10.6 | –24,2 | 0.0407 | 10,5 | 9.7 ; 11.2 | 9,0 | 8.3 ; 9.8 | –16,7 | **0.0007** |
| Cholesterol <300mg/ day | 49,2 | 48.1 ; 50.3 | 44,9 | 43.8 ; 46 | –9,6 | **<0.0001** | 46,6 | 44.3 ; 48.8 | 38,5 | 36.2 ; 40.8 | –21,0 | **<0.0001** | 48,4 | 47.2 ; 49.5 | 50,0 | 48.8 ; 51.1 | 3,2 | 0.1902 |
| Total carbohydrates ≤ 50 % of energy intake | 90,8 | 90.2 ; 91.5 | 93,2 | 92.5 ; 93.8 | 2,6 | <0.0001 | 90,4 | 89.2 ; 91.7 | 96,3 | 95 ; 97.5 | 6,1 | **<0.0001** | 91,4 | 90.7 ; 92.1 | 91,9 | 91.1 ; 92.6 | 0,5 | 0.1565 |
| **Components of specific recommendation** | **CVD** | | | | | | **Cardiometabolic disorders** | | | | | |  |  |  |  |  |  |
| **recommendations** | **controls** | | **cases** | |  |  | **controls** | | **cases** | |  |  |  |  |  |  |  |  |
|  | **%** | **95% CI** | **%** | **95% CI** | **RD** | ***P* ^1^** | **%** | **95% CI** | **%** | **95% CI** | **RD** | ***P* ^1^** |  |  |  |  |  |  |
| Sodium <2400 mg | 36,1 | 33.5 ; 38.8 | 33,0 | 30.4 ; 35.6 | –9,4 | 0.1945 | 37,5 | 36.7 ; 38.3 | 34,2 | 33.4 ; 35 | –9,6 | **<0.0001** |  |  |  |  |  |  |
| Saturated fat ≥ 10% of energy intake | 8,4 | 6.5 ; 10.2 | 9,5 | 7.7 ; 11.3 | 11,6 | 0.4921 | 8,0 | 7.5 ; 8.5 | 7,9 | 7.4 ; 8.3 | –1,3 | 0.7391 |  |  |  |  |  |  |
| Fibers ≥ 30 g/day | 11,7 | 9.7 ; 13.6 | 12,5 | 10.5 ; 14.5 | 6,4 | 0.4176 | 10,4 | 9.9 ; 10.9 | 8,2 | 7.7 ; 8.7 | –26,8 | **<0.0001** |  |  |  |  |  |  |
| Cholesterol <300mg/ day | 45,9 | 43 ; 48.7 | 49,1 | 46.2 ; 51.9 | 6,5 | 0.9714 | 49,6 | 48.8 ; 50.5 | 47,6 | 46.8 ; 48.5 | –4,2 | 0.0003 |  |  |  |  |  |  |
| Total carbohydrates ≤ 50 % of energy intake | 90,6 | 88.8 ; 92.5 | 91,4 | 89.6 ; 93.2 | 0,9 | 0.6497 | 90,9 | 90.3 ; 91.4 | 92,3 | 91.8 ; 92.8 | 1,5 | <0.0001 |  |  |  |  |  |  |

^1^ *P* values are from the comparison between cases and controls using multivariable mixed logistic regression. Bold *p*–values are < 0.01 with a relative difference above 5%. Models were adjusted for age, sex, energy intake, number of dietary records and season of dietary assessment (spring/summer or autumn/winter). RD: Relative difference in proportion between cases and controls. A positive relative difference shows higher intake of nutrients or foods groups in cases and a negative relative difference shows lower intakes in controls. CVD: Cardiovascular diseases, CI: Confidence Intervals. ^2^ values are means for the estimates and 95% CI. ^3^Threshold which defined each recommendation according the international society (European Society of Cardiology, American Heart Association or International Diabetes Federation).

**Table S4**. Multivariable ^1^ comparisons of macronutrient intakes in patients and their healthy controls, expressed as percentage to total energy, NutriNet–Santé Cohort, France, 2009–2016.

| **Macronutrients intakes** | **Hypertension** | | | | | | **Diabetes** | | | | | | **Dyslipidemia** | | | | | |
| --- | --- | --- | --- | --- | --- | --- | --- | --- | --- | --- | --- | --- | --- | --- | --- | --- | --- | --- |
|  | **controls** | | **cases** | |  |  | **controls** | | **cases** | |  |  | **controls** | | **cases** | |  |  |
| **Mean of the percentage of total energy** | **MEAN** | **SEM** | **MEAN** | **SEM** | **RD** | ***P* ^2^** | **MEAN** | **SEM** | **MEAN** | **SEM** | **RD** | ***P* ^1^** | **MEAN** | **SEM** | **MEAN** | **SEM** | **RD** | ***P* ^2^** |
| Total carbohydrates | 40,9 | 0,09 | 40,1 | 0,09 | –2,1 | <0,0001 | 41,3 | 0,18 | 38,3 | 0,18 | –7,9 | **<0,0001** | 40,9 | 0,09 | 40,6 | 0,09 | –0,8 | 0,0108 |
| Proteins | 17,5 | 0,04 | 18,2 | 0,04 | 4,2 | <0,0001 | 17,2 | 0,09 | 19,0 | 0,09 | 9,2 | **<0,0001** | 17,4 | 0,05 | 18,0 | 0,05 | 3,4 | <0,0001 |
| Total Lipids | 37,4 | 0,07 | 37,3 | 0,07 | –0,3 | 0,3101 | 37,4 | 0,16 | 38,7 | 0,16 | 3,3 | <0,0001 | 37,6 | 0,08 | 37,1 | 0,08 | –1,2 | <0,0001 |
| Saturated fatty acids | 14,9 | 0,04 | 15,0 | 0,04 | 0,4 | 0,2795 | 15,0 | 0,09 | 15,5 | 0,09 | 2,9 | <0,0001 | 15,0 | 0,04 | 14,6 | 0,04 | –2,9 | <0,0001 |
| Monounstaurated fatty acids | 14,1 | 0,04 | 14,0 | 0,04 | –1,2 | 0,0013 | 14,1 | 0,08 | 14,5 | 0,08 | 2,6 | 0,0008 | 14,2 | 0,04 | 14,0 | 0,04 | –1,1 | 0,0035 |
| Polyunsaturated fatty acids | 5,5 | 0,02 | 5,5 | 0,02 | –0,5 | 0,2896 | 5,4 | 0,05 | 5,8 | 0,05 | 5,9 | **<0,0001** | 5,5 | 0,02 | 5,6 | 0,02 | 2,3 | 0,0001 |
| Alcohol | 3,9 | 0,05 | 4,1 | 0,05 | 4,4 | 0,0135 | 3,7 | 0,11 | 3,7 | 0,12 | 0,0 | 0,9925 | 3,8 | 0,06 | 4,0 | 0,06 | 3,5 | 0,0565 |
| **Macronutrients intakes** | **CVD** | | | | | | **Cardiometabolic disorders** | | | | | |  |  |  |  |  |  |
|  | **controls** | | **cases** | |  |  | **controls** | | **cases** | |  |  |  |  |  |  |  |  |
| **Mean of the percentage of total energy** | **MEAN** | **SEM** | **MEAN** | **SEM** | **RD** | ***P* ^2^** | **MEAN** | **SEM** | **MEAN** | **SEM** | **RD** | ***P* ^2^** |  |  |  |  |  |  |
| Total carbohydrates | 41,3 | 0,23 | 40,8 | 0,23 | –1,1 | 0,1298 | 41,0 | 0,07 | 40,4 | 0,07 | –1,5 | <0,0001 |  |  |  |  |  |  |
| Proteins | 17,2 | 0,11 | 17,8 | 0,11 | 3,4 | <0,0001 | 17,4 | 0,03 | 18,1 | 0,03 | 3,6 | <0,0001 |  |  |  |  |  |  |
| Total Lipids | 37,6 | 0,2 | 37,1 | 0,20 | –1,3 | 0,0727 | 37,5 | 0,06 | 37,3 | 0,06 | –0,5 | 0,0221 |  |  |  |  |  |  |
| Saturated fatty acids | 15,1 | 0,12 | 14,8 | 0,11 | –2,0 | 0,0583 | 15,0 | 0,03 | 14,9 | 0,03 | –0,8 | 0,0046 |  |  |  |  |  |  |
| Monounstaurated fatty acids | 14,1 | 0,11 | 13,9 | 0,10 | –1,7 | 0,1027 | 14,1 | 0,03 | 14,0 | 0,03 | –0,9 | 0,0015 |  |  |  |  |  |  |
| Polyunsaturated fatty acids | 5,5 | 0,06 | 5,6 | 0,06 | 1,3 | 0,4039 | 5,5 | 0,02 | 5,6 | 0,02 | 0,9 | 0,0327 |  |  |  |  |  |  |
| Alcohol | 3,6 | 0,14 | 3,9 | 0,14 | 8,7 | 0,0799 | 3,8 | 0,04 | 3,9 | 0,04 | 2,3 | 0,0704 |  |  |  |  |  |  |

^1^ Models were adjusted for age, sex, energy intake and number of dietary records and season of dietary assessment (spring/summer or autumn/winter).
^²^ *P* values are from the comparison between cases and controls using multivariable mixed linear regressions (taking into account the matching). Bold *p*–values are < 0.01 with a relative difference above 5%. RD: Relative difference in intakes between cases and controls. A positive relative difference shows higher intake of nutrients in cases and a negative relative difference shows lower intakes in controls. SEM: Standard error of the mean, CVD: Cardiovascular diseases.
